# SW-Net: A Direction-Aware Deep Learning Model for Shipwreck Segmentation in Side-Scan Sonar Imagery

**DOI:** 10.3390/s26113483

**Published:** 2026-06-01

**Authors:** Jiani Dai, Jie He

**Affiliations:** The School of Architecture, Harbin Institute of Technology, Shenzhen 518055, China; daijiani@hit.edu.cn

**Keywords:** marine archaeology, side-scan sonar imagery, image segmentation, shipwreck detection, directional filter bank

## Abstract

**Highlights:**

**What are the main findings?**

- To address the distinct geometric characteristics and scale variations in shipwreck targets, SW-Net was proposed as a specialized encoder–decoder architecture that fuses high-level semantic context with fine-grained spatial details through a multi-scale input module and refined skip connections.
- To better capture complex shapes, a directional filter bank and a directional attention mechanism are introduced. Steerable Gaussian kernels are used to extract structural boundaries, while orientation-specific features are adaptively weighted to reduce the effects of reverberation.

**What are the implications of the main findings?**

- Embedding geometric constraints and directional priors into lightweight architectures proves more effective than increasing model depth for distinguishing man-made targets from seabed backgrounds.
- The computational efficiency of SW-Net enables real-time deployment on resource-constrained autonomous underwater vehicles for “search-and-inspect” missions, reducing the labor and costs of large-scale surveys.

**Abstract:**

Side-scan sonar is a critical instrument for underwater cultural heritage preservation, as it allows large-scale detection of shipwrecks in turbid waters where optical methods fail. However, the automated segmentation of these targets remains a significant challenge, as severe speckle noise and complex seabed reverberations often obscure the distinctive geometric features of submerged structures. To address this challenge, this paper proposes SW-Net, which utilizes a multi-scale input strategy and a novel Directional Filter Bank to inject physical priors into the feature extraction process. Furthermore, by coupling this with a directional attention mechanism, the network dynamically modulates structural features to accurately segment targets despite intensity inversions and speckle noise. As demonstrated by the experimental results on the AI4Shipwrecks dataset, the SW-Net outperforms seven representative segmentation architectures, achieving the highest intersection over union of 39.43% and an F1-score of 56.56%. In addition, the model exhibits superior robustness against complex seabed interference while maintaining the lowest computational complexity of 4.01 million parameters among the evaluated methods. Taken together, the SW-Net is proposed to offer a practical solution for shipwreck detection on resource-constrained autonomous underwater vehicles.

## 1. Introduction

Oceans hide a treasure trove of heritage such as shipwrecks, but they are in danger due to looting, the exploitation of marine resources, climate disruption, and pollution [1]. The United Nations Educational, Scientific and Cultural Organization (UNESCO) proposed the 2001 Convention on the Protection of the Underwater Cultural Heritage to safeguard submerged cultural resources and ensure their preservation [2]. However, current mapping and characterization of the seabed remain significantly less comprehensive than those of terrestrial landscapes [3]. The seabed information deficit can lead to critical issues, such as navigation hazards arising from uncharted obstructions and the inadvertent destruction of unmapped historical sites during trawling or construction [4]. The contradiction between the increasing demand for offshore expansion and the constraints of underwater environmental protection is becoming increasingly apparent [5]. Therefore, an efficient and reliable target segmentation method is needed, as it can help acquire information on the distribution of shipwrecks, examine their preservation status, and support restoration and protection efforts.

Shipwreck segmentation faces multiple challenges, such as missed segmentation and false segmentation due to blurred outlines or complex seabed backgrounds [6,7]. At present, there are two main ways of obtaining shipwreck data. One is through manual investigation, which is generally carried out by professional divers or manned submersibles [8]. This approach takes heavy workloads, poses severe safety risks due to pressure and decompression sickness, has depth limitations, and cannot survey vast areas of the ocean floor in real time. Therefore, it is imperative that modern technologies are used efficiently to fulfill the demands of monitoring risks to shipwrecks [9]. The other is through optical or acoustic remote sensing technology [10]. Optical remote sensing images are outstanding in performing high-resolution visual inspection at close range. But in the beginning, the cost is very high, and underwater optical images are often degraded by turbidity, light attenuation, and scattering; thus, it is hard to use this method to detect and surveil shipwrecks over large-scale turbid waters [11]. In recent years, side-scan sonar (SSS), as an acoustic remote sensing technology mounted on autonomous underwater vehicles (AUVs) or tow fish, has been widely applied in the fields of marine archaeology, pipeline inspection [12], military mine countermeasures [13], and disaster search and rescue, in light of its acoustic imaging capabilities, wide swath coverage, and ability to penetrate dark or turbid waters [14,15]. In addition, it can be used and customized freely according to different operating frequencies and tow heights. Therefore, it is preferable to allow the SSS-equipped platform to perform image acquisition and data collection in the target sea area, thereby enabling the acquisition of higher-quality acoustic data, which will facilitate the subsequent identification of the required shipwreck features.

Despite the efficient data acquisition capabilities of SSS, the automatic interpretation of these acoustic images remains a formidable bottleneck. Compared with optical photography, SSS imagery is fundamentally generated by the interplay of echo intensity and time-of-flight, resulting in data that are plagued by severe multiplicative speckle noise, geometric distortions [16], and uneven grayscale [17]. For instance, in complex seabed environments, the acoustic return from a corroded shipwreck hull is often indistinguishable from that of large rock formations or sand ripples due to low contrast and signal scattering. Furthermore, targets are frequently obscured by acoustic shadows or sediment accumulation [18], making boundary delineation notoriously difficult. Traditional segmentation algorithms based on thresholding or clustering, such as K-means or Markov random fields [19], often fail in these scenarios because they cannot accurately delineate objects in underwater sonar images characterized by complex textural dependencies in the sonar data [20]. Similarly, standard deep learning models designed for terrestrial optical imagery, such as the vanilla U-Net [21] or fully convolutional network (FCN) [22], still struggle to capture high-frequency edge details in the presence of heavy speckle noise. Recent studies have shown that applying these generic networks directly to SSS data often results in fragmented segmentation masks, in which the continuous structure of a wreck is broken into disjointed blobs, thereby losing critical structural integrity [23].

To address these persistent obstacles in underwater acoustic perception, a novel deep learning framework tailored for shipwreck segmentation is presented in this study. Recognizing that standard optical-based networks often struggle with the distinct geometric characteristics and scale variations in sonar targets, a specialized encoder–decoder architecture, designated as SW-Net, where SW stands for shipwrecks, is constructed to refine feature fusion and bridge the semantic gap between encoding and decoding stages. Unlike conventional U-Net variants that rely solely on unconstrained, data-driven learning, the core novelty of SW-Net lies in proposing a physics-guided deep learning paradigm. By deeply coupling traditional acoustic physical priors with dynamic neural attention mechanisms, this architecture effectively constrains the feature learning process, preventing the highly flexible network from overfitting to the severe speckle noise inherent in sonar data. The primary contributions of this research are summarized as follows:

(1) A specialized encoder–decoder architecture, designated as SW-Net, is constructed for shipwreck segmentation in side-scan sonar imagery. Built upon a U-Net-like backbone, the model integrates multi-scale input processing, refined skip connections, and an offset convolution module with fixed asymmetric padding to better capture scale variations and irregular shipwreck boundaries.
(2) A directional filter bank (DFB) is proposed to inject physical prior knowledge into feature extraction. Based on fixed Gaussian derivative kernels, the DFB decomposes features into directional responses, helping the network distinguish meaningful structural edges from speckle noise, acoustic shadows, and seabed reverberation.
(3) A directional attention mechanism (DAM) is developed to adaptively weight orientation-specific features extracted by the DFB. By emphasizing discriminative structural directions and guiding the subsequent offset convolution, DAM enhances the representation of complex shipwreck morphologies under low-contrast and noisy sonar conditions.

## 2. Related Work

### 2.1. Semantic Segmentation of Side-Scan Sonar Imagery

Semantic segmentation of SSS imagery presents a formidable challenge in marine exploration, primarily attributable to the inherent acoustic characteristics of the sensor, such as severe speckle noise, intensity inhomogeneity, and extreme class imbalance between small targets and the vast seabed background [24,25]. Driven by the operational necessity for deployment on AUVs, earlier architectural paradigms prioritized computational efficiency [26]; notably, RT-Seg [27] and ECNet [24] employed lightweight, depth-wise separable convolutions to enable real-time processing rates.

However, owing to the limitations of standard convolutional neural networks (CNNs) [28,29] in capturing global context, the field has recently witnessed a shift towards hybrid architectures. In response to this limitation, recent state-of-the-art models, such as CGF-U-Net [30] and SonarNet [31], have incorporated Transformer blocks to enhance global feature extraction. Similarly, the cross-scale feature interaction network (CSFINet) [32] addresses feature loss through multiscale interaction. Nevertheless, despite these advancements, a critical limitation remains: these models largely treat spatial features isotropically. As has been observed in similar synthetic aperture radar (SAR) tasks, man-made targets exhibit distinct geometric properties, such as straight edges and regular shapes, that distinguish them from natural backgrounds [33,34,35]. In contrast, current SSS models often fail to explicitly leverage these geometric priors, treating the random texture of the seabed and the structured edges of a wreck with the same convolutional logic, which leads to boundary blurring under low-contrast conditions.

### 2.2. Attention Mechanisms in Computer Vision

Attention mechanisms have been introduced to enhance the representational power of CNNs by enabling networks to focus on informative features while suppressing irrelevant ones [36,37]. General attention modules typically operate across channel and spatial dimensions. The Squeeze-and-Excitation block pioneered channel attention by explicitly modeling inter-channel dependencies [38], while the convolutional block attention module (CBAM) integrated spatial attention to guide the network on what to look at and where to look [39].

However, a significant misalignment arises when applying these generic mechanisms to shipwreck segmentation, as off-the-shelf computer vision models often struggle with domain-specific patterns. In response to this, fine-tuning is effective in improving CNN transferability and can provide remarkable accuracy that outperforms previous state-of-the-art methods [40]. Recent lightweight and hybrid segmentation models have further attempted to improve feature representation through adaptive feature modulation and attention-based context modeling. For example, LHNet combines CNN-based spatial detail extraction with Transformer-based global dependency modeling through multi-scale sliding window attention [41]. TriEncoderNet further integrates CNN, Transformer, and HOG-based encoders, using attention-based fusion to combine local, global, and edge-related features for challenging underwater sonar segmentation [42]. Nevertheless, standard attention modules [38,39] are largely orientation-agnostic. These modules tend to enhance features driven by activation intensity while lacking explicit mechanisms to model spatial alignment. As a result, in sonar imagery, strong echoes may come from either irregular rock formations or man-made hulls, so relying only on intensity-based attention is not enough. These methods therefore struggle to capture orientation-related features, such as linearity and continuity, that help distinguish structural edges from background reverberation.

### 2.3. Directional Feature Learning

Directionality is a fundamental attribute of visual perception and is essential for distinguishing the regular geometric structures of man-made objects from natural backgrounds [43]. While early computer vision explicitly modeled orientation through hand-crafted descriptors [44], most modern deep learning frameworks rely on the implicit learning of orientation-sensitive features via convolutional kernels, rather than explicit orientation encoding [21,22,45]. To mitigate the inefficiency arising from learning multiple transformed instances of the same feature, recent research has increasingly focused on transformation-equivariant network designs [46,47]. However, these methods often incur a heavy computational burden, making them unsuitable for real-time AUV applications.

In the realm of attention mechanisms, methods such as coordinate attention [48], large selective kernel (LSK) networks [49], and deformable convolutions [50] have begun to explore dynamic spatial context. Deformable convolutions dynamically learn spatial offsets based on input features to adapt to target shapes. While this unconstrained spatial adaptation offers high flexibility in standard optical computer vision, it lacks explicit feature stacking constraints. Consequently, when applied to side-scan sonar imagery, these purely data-driven methods are highly susceptible to severe speckle noise and complex reverberations. They often fit random acoustic noise rather than the actual structural boundaries of shipwrecks.

Moreover, few mechanisms are specifically optimized to enhance the sharp, linear edges characteristic of targets in noisy environments. Drawing inspiration from shape-constrained segmentation in SAR imagery [51], where prior geometric knowledge is incorporated to overcome noise, there is a clear need for a lightweight mechanism that explicitly perceives features along critical directions. This need motivates the design of our DAM, which aims to distinguish structural anomalies from the fractal-like speckle noise of the seabed without the overhead of full rotational equivariance.

## 3. Method

### 3.1. Framework of the SW-Net

**Figure 1:**
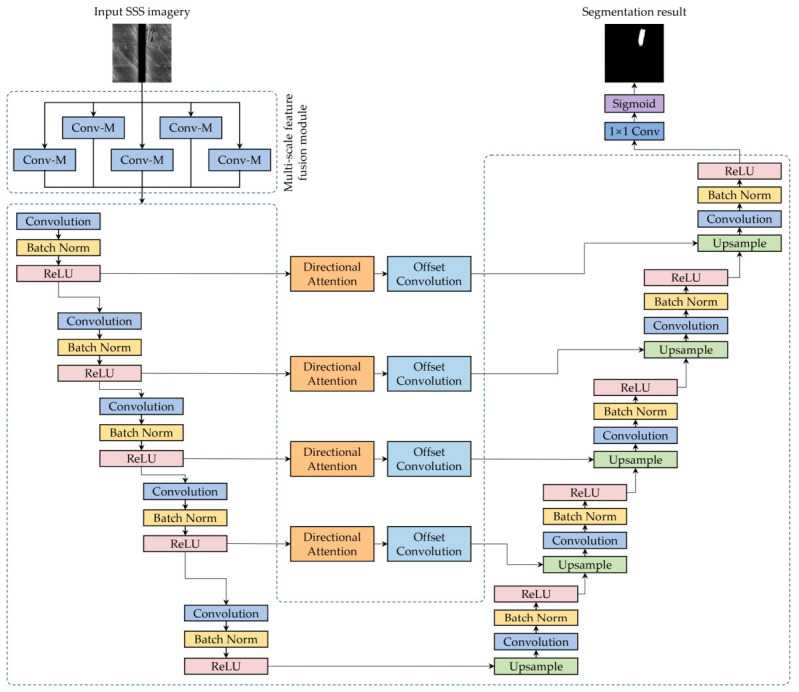
Framework of the SW-Net.

The overall network architecture of the proposed SW-Net is illustrated in Figure 1. Constructed upon a U-Net-like backbone, the SW-Net is specifically engineered to address the distinct geometric characteristics of shipwreck targets in SSS imagery. The network follows an encoder–decoder design paradigm, facilitating the simultaneous extraction of high-level semantic context and the preservation of low-level spatial details. To accommodate the varying scales of underwater targets, a multi-scale input processing module is employed at the initial stage. The input image is processed in parallel by multiple convolutional branches, each configured with distinct kernel sizes and dilation rates. These multi-scale features are subsequently concatenated and fused to form a rich initial feature representation.

The encoder path consists of five hierarchical stages, where downsampling is performed via max-pooling operations to progressively expand the receptive field and abstract semantic features. A distinguishing improvement of the SW-Net is the sophisticated refinement applied to the features within the skip connections. While the general U-Net-like structure with skip connections is a standard paradigm in image segmentation, our architectural novelty lies in fundamentally repurposing these connections for acoustic data. Rather than performing a simple feature concatenation or employing generic attention modules, we transform the skip connections into a physics-guided refinement pipeline. Compared with the standard U-Net architectures that directly transfer encoder features to the decoder, the proposed model introduces a two-step enhancement strategy to bridge the semantic gap. Feature maps from the encoder are first processed by the directional attention mechanism, as detailed in Section 3.3, to explicitly highlight orientation-specific structural information and suppress noise.

**Figure 2:**
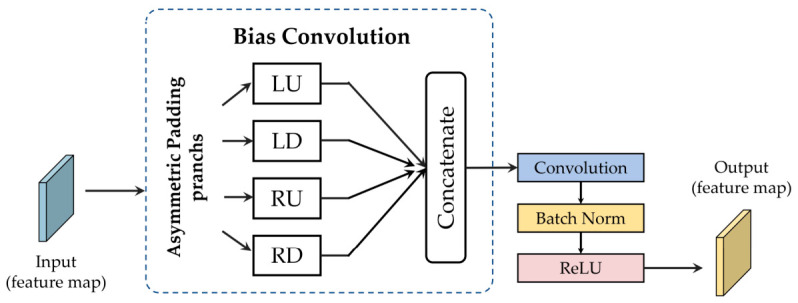
Structure of the offset convolution.

Following the directional attention, the features are further processed by an offset convolution module proposed by [52]. The offset convolution is designed to capture geometric deformations and irregular boundaries inherent to shipwreck structures. As shown in Figure 2, the Offset Convolution module consists of four parallel convolutional branches, each configured with asymmetric reflection padding on a distinct pair of adjacent sides: left and up (LU), left and down (LD), right and up (RU), and right and down (RD). It should be noted that, unlike deformable convolutional networks which rely on a learnable prediction head to generate dynamic per-pixel spatial offsets, the spatial offsets in this module are structurally fixed through the padding configuration and are not updated during training. The convolutional kernel weights within each of the four directional branches remain fully learnable. The feature maps produced by these branches are concatenated and fused through a 1 × 1 convolution, batch normalisation, and a ReLU activation, allowing the network to adaptively aggregate information from the four predetermined geometric perspectives without the computational burden of dynamic offset prediction.

### 3.2. Directional Filter Bank

**Figure 3:**
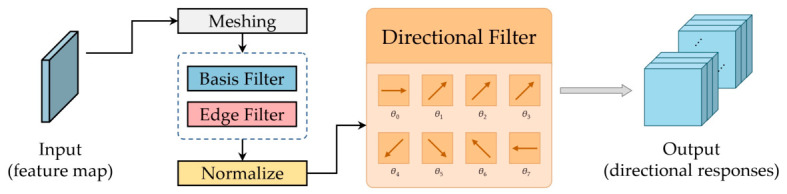
The structure of the directional filter bank.

Although SSS imagery poses difficulties for semantic segmentation because of heavy speckle noise, targets such as shipwrecks often show clear geometric structures. These structures usually form strong edges between bright echoes and acoustic shadows. Standard CNNs initialize kernels randomly, meaning they lack structured feature extraction capabilities at the start. To address this limitation, the DFB is proposed, as shown in Figure 3. It is important to note that while steerable filters and Gabor-like directional filters have been utilized in traditional sonar image processing for decades, they have conventionally been restricted to isolated pre-processing steps or static, shallow feature extractors. Our novelty does not lie in the invention of steerable filters, but rather in embedding them directly into a deep convolutional framework as a structural constraint module. By injecting this fixed, mathematically defined physical prior into the network, the DFB acts as a deterministic anchor that prevents the highly flexible learnable layers from overfitting to the chaotic speckle noise, thereby extracting robust edge features.

The DFB is built upon the theory of steerable filters. The first derivative of a Gaussian function is utilized as the core kernel. The Gaussian component smooths out speckle noise [52,53], while the derivative operation acts as an edge detector [54]. A standard 2D Gaussian function G(x,y) with a scale σ is defined as:

(1) $$G(x,y)=\;exp(-\frac{x^{2}+y^{2}}{2\sigma^{2}}),$$

(2) $$x,y\in Z,-\left\lfloor \frac{s}{2}\right\rfloor \leq x,y\leq \left\lfloor \frac{s}{2}\right\rfloor ,$$

where x and y represent the pixel coordinates, and s represents the kernel size of the Gaussian function.

Based on function (1) and (2), two basis filters are generated. These correspond to the derivatives in the horizontal $G_{0}(x,y)$ and vertical $G_{90}(x,y)$ directions. They represent the fundamental components of any edge:

(3) $$G_{0}(x,y)=\;-\frac{x}{\sigma^{2}}G(x,y),$$

(4) $$G_{90}(x,y)=-\frac{y}{\sigma^{2}}G(x,y).$$

A key advantage of this approach is computational efficiency. Physical rotation of the input image or expensive interpolation is not required. Instead, an edge filter $G_{\theta }(x,y)$ at an arbitrary orientation θ is synthesized linearly. It is formed by a weighted combination of the two basis filters. The steering formula is defined as:

(5) $$G_{\theta }(x,y)=\;cos(\theta )G_{0}(x,y)+sin(\theta )G_{90}(x,y).$$

In the implementation, a bank of K filters is generated. These filters cover discrete orientations uniformly distributed from 0 to π. The weights are registered as non-trainable buffers in the model. They remain fixed during the training process, providing a stable feature extraction mechanism. Normalization is also applied to each kernel. The mean is subtracted to ensure a zero sum which causes the filter response to approximate zero in flat or homogeneous regions. As a result, the module extracts significant structural and directional responses.

During the forward pass, the input feature map X of shape (C, H, W) is processed by the DFB. The filter bank is applied to every channel of the input independently using grouped convolutions. For each input channel, K directional response maps are produced. The final output is a 4D tensor of shape (C, K, H, W). This representation decomposes the visual information into specific directional components. Consequently, subsequent attention mechanisms are enabled to identify exactly which direction contains the most relevant structural information.

### 3.3. Directional Attention Mechanism

**Figure 4:**
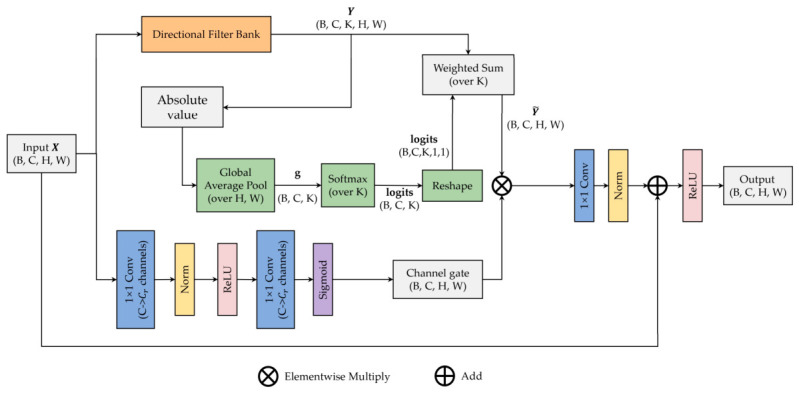
The structure of the directional attention mechanism.

To enhance feature representation by explicitly capturing orientation-specific information, the directional attention mechanism is proposed, as shown in Figure 4. The core innovation of our solution emerges from the deep synergy between the DAM and the DFB, rather than simply replacing a standard attention module. Standard attention mechanisms operate on unconstrained, purely data-driven feature maps, which often fail in sonar imagery due to severe noise corruption. In contrast, the DAM is explicitly designed to act as a dynamic decoder for the deterministic physical priors provided by the DFB. Compared with standard convolutions, which treat spatial directions uniformly, this module dynamically aggregates features from multiple orientations based on their saliency and modulates them with global context.

X is a matrix with shape (B, C, H, W) and is denoted as the input feature map. First, the input is processed by a DFB to yield directional responses Y with shape (B, C, K, H, W), where K represents the number of orientation channels.

To determine the importance of each direction, the absolute magnitude of the responses, |Y|, is computed to ensure robustness against intensity inversions. Then global average pooling is applied across the spatial dimensions (H, W) to obtain a descriptor $g_{b,c,k}$. The descriptor for the b-th batch, c-th channel, and k-th direction is calculated as:

(6) $$g_{b,c,k}=\;\frac{1}{H\times W}\;\sum_{i=1}^{H}\sum_{j=1}^{W}\left|Y_{b,c,k,i,j}\right|.$$

Subsequently, the attention weights α are derived by applying a softmax function along the directional dimension K:

(7) $$\alpha_{c,k}=\;\frac{exp(g_{c,k})}{\sum_{j=1}^{K}exp(g_{c,j})}.$$

These weights are used to aggregate the original signed directional responses Y. The weighted sum produces a directionally fused feature map $\tilde{Y}$.

(8) $${\tilde{Y}}_{b,c,i,j}=\sum_{k=1}^{K}{\alpha_{b,c,k}\cdot Y}_{b,c,k,i,j}$$

To further improve feature representation, a channel-wise gating mechanism based on the original input X is employed. The gate is a lightweight bottleneck architecture designed to capture channel dependencies with controlled complexity. Specifically, the channel dimension is compressed by a reduction ratio r and then expanded back to C, followed by a Sigmoid activation to generate a feature map. The intermediate channel size is defined as:

(9) $$C_{r}=\;\left\lfloor \frac{C}{r}\right\rfloor .$$

The aggregated features $\tilde{Y}$ are modulated by this gate. Finally, the module utilizes a residual connection. The modulated features are fused with the original input X through a 1 × 1 convolution and normalization, ensuring stable gradient propagation.

From the perspective of training dynamics, the sequential arrangement of the fixed-weight DFB within the DAM and the learnable offset convolution forms an end-to-end differentiable pipeline. The DFB is embedded as a sub-module inside the DAM, where it serves as the first stage of directional feature extraction. During the forward pass, the encoder features are first processed by the DAM, whose learnable components include the channel-gating bottleneck and the residual fusion convolution. The output of the DAM is then fed into the offset convolution module. During backpropagation, the loss gradient updates the convolutional weights of the offset convolution and the learnable components of the DAM. When the gradient reaches the DFB, the fixed Gaussian derivative kernels, registered as non-trainable buffers, are not modified. Nevertheless, because the grouped convolution operation that applies these kernels is differentiable, the gradient propagates through the DFB via the chain rule and continues into the preceding encoder stages. This design ensures that while the DFB provides a stable, physically grounded directional prior, the encoder is actively guided by the gradient signal to produce feature maps that align optimally with this directional decomposition, and the downstream modules adapt their learned weights to the resulting directionally enhanced representations.

## 4. Experiments and Results

This section presents the dataset, experimental details, ablation results, and comparative results.

### 4.1. Dataset Preparation

The raw SSS imagery used in this study is sourced from the open-access AI4Shipwrecks dataset [55]. To prepare high-quality inputs suitable for deep learning model processing, the original full sonar images from different survey sites were first converted into grayscale and preprocessed using a Gaussian filter [56]. Then, the images were segmented into standardized samples. Every sample is set to a size of 1024 × 1024 pixels. If there are any samples smaller than this size, padding, labeled as 0, will be added to make them become the same size as the other samples. As for the corresponding label masks, the shipwreck regions were marked as 1, whereas the background regions were labeled as 0.

In the original SSS imagery, shipwreck pixels account for only a very small portion of the image area. Even in samples containing shipwreck targets, the average proportion of target pixels is approximately 0.8%. Such a strong class imbalance makes it difficult for the model to learn sparse target features effectively. To address this issue, a targeted cleaning strategy was implemented for the training set. Specifically, we define images containing only flat, featureless seabed or the nadir gap directly beneath the sonar—without any rocks or complex topologies—as easy negative samples. While the vast majority of these redundant easy negative samples were manually excluded to prevent the model from overfitting to the background, we strictly ensured that at least one representative pure background image was retained for each survey site.

The theoretical basis for this operation is that conventional background features are already sufficiently represented in the backgrounds of images containing shipwreck targets, and the intentionally retained pure background samples provide adequate baseline features for the model to recognize normal seabed. Eliminating the massive redundancy of identical flat patches significantly enhances the learning efficiency regarding critical shipwreck features and saves computational resources. Furthermore, to ensure sample diversity and accurately simulate real-world wide-area inspections during evaluation, all images in the test set were completely retained without any exclusion. In the end, 1532 training samples were obtained from 14 survey sites, while the test set comprises 1722 samples derived from a separate set of 15 survey sites.

### 4.2. Experimental Details

The experiments were conducted across two hardware environments, both running on the Ubuntu 20.04 operating system. The primary setup utilized four NVIDIA RTX A6000 GPUs, implemented with PyTorch 1.13.1 and CUDA 11.7. Additionally, a cloud server equipped with NVIDIA RTX 5090 GPUs (NVIDIA, Santa Clara, CA, USA), utilizing PyTorch 2.8 and CUDA 12.8 support, was employed to complete partial training tasks. The Adam optimizer was used for training with a learning rate of 0.001, and the batch size was 4 in all experiments. To rule out randomness and ensure reliability, every reported IoU and F1-score is a statistical result (mean and standard deviation) derived from five independent repeated experiments. Furthermore, the inference time and frames per second (FPS) are statistical results calculated based on the time required to process 1000 images on a single RTX A6000 GPU. The BCE-Dice loss function is used for training all models and defined as follows:

(10) $$L_{total}=\;L_{BCE}+L_{Dice},$$

(11) $$L_{BCE}=-\frac{1}{N}\sum_{i=1}^{N}\left[g_{i}\log\left(p_{i}\right)+(1-g_{i})log(1-p_{i})\right],$$

(12) $$L_{Dice}=1-\frac{2\sum_{i=1}^{N}p_{i}g_{i}}{\sum_{i=1}^{N}p_{i}+\sum_{i=1}^{N}g_{i}},$$

where N denotes the total number of pixels in the image, $p_{i}$ represents the predicted probability of the *i*-th pixel belonging to the shipwreck class, and $g_{i}$ is the corresponding ground truth label. By minimizing this joint loss, the network is explicitly guided to accurately segment the minority shipwreck targets while maintaining training stability.

### 4.3. Metrics

To quantitatively compare SW-Net with other models, an evaluation framework based on pixel-level classification accuracy is employed. The evaluation is based on the confusion matrix, which classifies each pixel prediction into four categories: True Positive (TP), False Positive (FP), True Negative (TN), and False Negative (FN). TP represents pixels correctly identified as part of the shipwreck target. FP represents background pixels, e.g., seabed, rocks, or water column, incorrectly classified as shipwreck. TN represents background pixels correctly identified as background. FN represents actual shipwreck pixels that the model failed to detect. In the terminology of classical pattern recognition, FP corresponds to a Type I error, in which background pixels are falsely identified as shipwreck targets, whereas FN corresponds to a Type II error, in which actual shipwreck pixels are missed.

Based on these fundamental components, intersection over union (IoU) and F1-score are used to evaluate the segmentation quality. IoU is a standard metric in semantic segmentation that measures the overlap between the predicted segmentation mask and the ground truth mask. It is calculated as the ratio of the area of intersection to the area of the union of the predicted and ground truth regions. The formula of IoU is defined as:

(13) $$\mathrm{IoU}\;=\;\frac{TP}{TP+FP+FN}.$$

The F1-score is similar to IoU, ranging from 0 to 1, with 1 indicating perfect overlap. It is often preferred when the data are imbalanced, which is common in sonar imagery because shipwreck targets occupy a much smaller area than the surrounding seabed background. By doubling the weight of TP, the F1-score provides a sensitive measure of how well the model captures the specific target features. The calculation of the F1-score is given by:

(14) $$F1-\mathrm{score}\;=\;\frac{2TP}{2TP+FP+FN}.$$

In the following experimental results, both IoU and F1-score are utilized to ensure a comprehensive evaluation. While IoU provides a robust measure of overall geometric alignment, F1-score offers insight into the model’s precision and sensitivity regarding the target structure.

### 4.4. Performance Comparison

To validate the effectiveness of the proposed SW-Net for automated shipwreck detection, this section presents a performance evaluation combining both quantitative metrics and qualitative visual analysis. The SW-Net is benchmarked against seven established segmentation architectures—U-Net [21], SegNet [45], Attention U-Net [57], UNet++ [53], MDOAU-Net [52], LHNet [41], and TriEncoderNet [42]—to assess its capability in handling low-contrast SSS imagery. The evaluation first focuses on statistical performance indicators, specifically IoU and F1-score, to quantify the segmentation precision and recall. Subsequently, the analysis is extended to a visual inspection of segmentation results across various marine environments. This includes scenarios characterized by different degrees of target integrity, seabed reverberation, sediment occlusion, and blind zone interference, providing an assessment of the model’s robustness and generalization ability in practical underwater archaeological surveys.

#### 4.4.1. Metrics Comparison

A quantitative comparison between SW-Net and seven other models is presented in Table 1. To ensure statistical reliability, all models were evaluated across five independent experimental runs. The table reports the mean and standard deviation (std) for both the IoU and the F1-score derived from five independent runs. The proposed SW-Net achieves the best overall performance with the highest mean IoU of 39.43% and the highest mean F1-score of 56.56%.

Compared with classic architectures like SegNet and the original U-Net, SW-Net handles the complex outlines of shipwreck targets much more effectively in low-contrast side-scan sonar imagery. The standard U-Net typically suffers from serious over-segmentation and produces a massive number of false-positive pixels. This phenomenon suggests that the classic U-Net has difficulty distinguishing real shipwreck structures from seabed reverberations. By contrast, SW-Net successfully maintains a better balance between sensitivity and precision. It significantly reduces misclassified background pixels while maintaining a high true positive rate. This highlights the stronger robustness of the proposed model against noise and its ability to generate cleaner segmentation boundaries.

Furthermore, SW-Net outperforms advanced U-Net variants and recently proposed state-of-the-art models including MDOAU-Net, LHNet, and TriEncoderNet. Although UNet++ and Attention U-Net show marginal improvements over the baseline, they fail to suppress false detections as effectively as the proposed method. The recently introduced TriEncoderNet and the advanced MDOAU-Net exhibit a strong ability to reduce noise. However, SW-Net still outperforms them by maximizing true positives while effectively suppressing false alarms. The low standard deviations across the five independent runs further confirm the stability of SW-Net. These results validate that SW-Net provides the most effective balance of precision and recall, making it a highly dependable tool for automated shipwreck detection.

#### 4.4.2. Visualization Results Comparison

**Figure 5:**
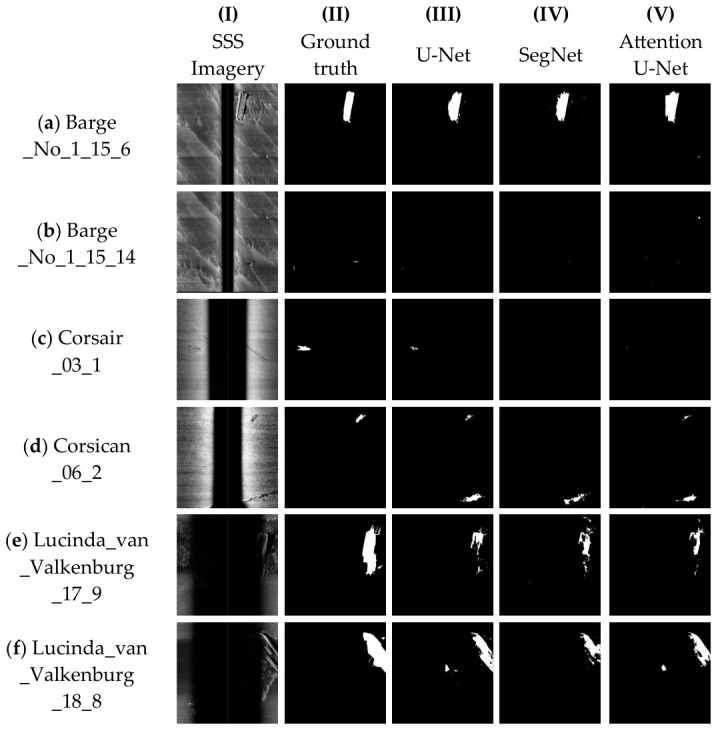

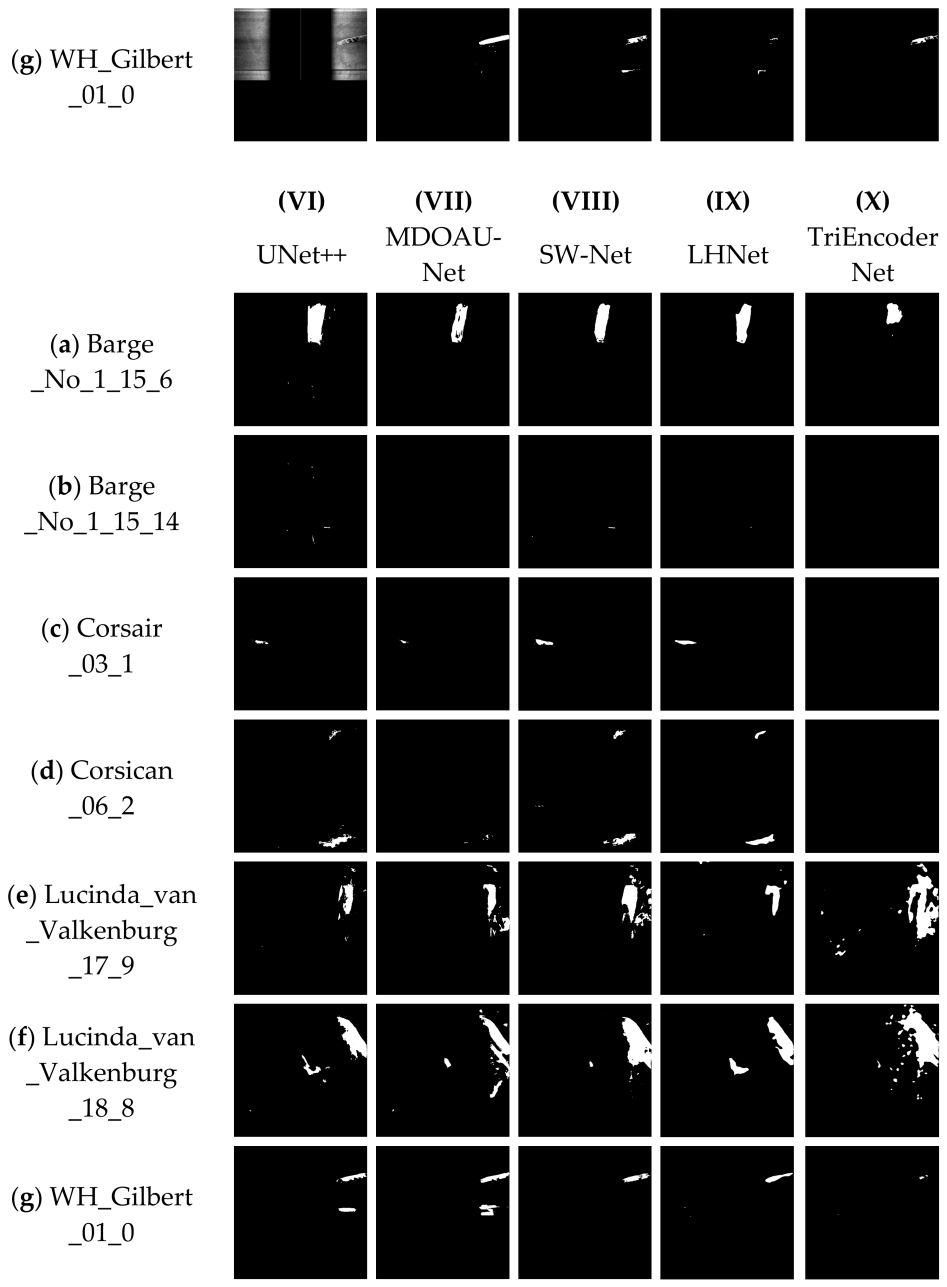
Segmentation results of different models.

Based on the comparison of the six sets of experimental images, as shown in Figure 5, a qualitative analysis of the visualization results is presented. This analysis encompasses various seabed environments, ranging from clear targets to complex backgrounds, and extending to scenarios involving occlusion and blind zone interference.

First, scenarios are analyzed where the shipwrecks exhibit relatively clear acoustic features and are not buried. Taking images Barge_No_1_15_6 and WH_Gilbert_01_0 as examples, the structures in these samples are reasonably intact. When processing the Barge sample, the SW-Net demonstrated superior completeness, accurately outlining the overall contour of the shipwreck. Conversely, masks generated by other models often exhibited fragmentation or gaps within the shipwreck’s interior. In the WH_Gilbert sample, although most models successfully detected the target’s presence, the MDOAU-Net, the UNet++, the SegNet, and the U-Net all suffered from over-segmentation, erroneously identifying non-shipwreck areas as targets. While the Attention U-Net identified the outer contour reasonably well, it lacked the internal detail captured by the SW-Net. The SW-Net accurately captured textural changes within the shipwreck, thereby avoiding missed detections caused by structural complexity.

Secondly, the robustness of the models is examined in complex backgrounds, particularly when the seabed contains rocky interference with acoustic features similar to shipwrecks. In image Barge_No_1_15_14, the target consists of two extremely small debris fragments, one of which is a partially buried bow. In this scenario, the MDOAU-Net failed completely and could not recognize the target. While the SegNet and the U-Net detected potential targets, they failed to accurately segment the contours and missed parts of the wreckage. Furthermore, the UNet++ and the Attention U-Net struggled to distinguish interference, mistaking surrounding rocks for the shipwreck. Only the SW-Net successfully excluded the rocks and accurately pinpointed the shipwreck’s location. A similar phenomenon occurred in Corsican_06_2, where the hull is damaged and lying on its side. The MDOAU-Net and the SegNet missed the target again, whereas the SW-Net provided the most complete contour recovery. However, it is worth noting that due to the high textural similarity between the rocks and the wreck, all models, including SW-Net, misclassified some large rocks as shipwreck parts, indicating that this specific scenario remains challenging.

Finally, extreme cases involving sediment occlusion and targets located in the nadir gap are analyzed. In Corsair_03_1, where half of the shipwreck is buried by sand, segmentation is extremely difficult. The SegNet failed to identify the target, and while Attention U-Net, MDOAU-Net, and U-Net detected the shipwreck, they could not reconstruct its shape. Under these occluded conditions, the UNet++ and the SW-Net performed best, with SW-Net still yielding a relatively complete contour. Regarding large shipwrecks located in the nadir gap, such as the Lucinda_van_Valkenburg series, i.e., samples 17_9 and 18_8, all models exhibited varying degrees of structural omission. However, a horizontal comparison shows that SW-Net had the fewest omissions and preserved the main structure of the shipwreck to the greatest extent. Although the SW-Net misclassified some noise or sand within the blind zone as shipwreck parts, this error is acceptable. Since the nadir gap is a fixed geometric region in sonar imagery, specific false positives generated within this area can be easily corrected through post-processing techniques. Therefore, taken as a whole, the SW-Net demonstrated optimal segmentation performance across various complex operating conditions.

### 4.5. Ablation Experiments

To systematically evaluate the contribution of each component, several distinct model variants were constructed. The standard U-Net was utilized as the baseline model, where standard skip connections were employed to concatenate encoder and decoder features. Subsequently, the OU-Net variant was created by replacing these standard skip connections with offset convolutions. This modification was aimed at testing the capability of deformable operations to handle the irregular shapes of shipwrecks. Building upon the OU-Net, the MDOAU-Net was developed by introducing two significant enhancements: a multi-scale feature fusion module to capture context at various resolutions, and a standard attention mechanism to process the input logits before they were passed to the offset convolution layers. Additionally, to verify the individual impacts of specific modules, U-Net + DFB and U-Net + DAM were also evaluated. Finally, the proposed SW-Net was established as the ultimate architecture. While the multi-scale fusion from MDOAU-Net was retained, the attention strategy was refined in this model. Specifically, the vanilla attention mechanism was replaced by an offset convolution with a DAM. This design choice was intended to more effectively guide the deformable sampling process.

The analysis begins by examining the transition from the baseline U-Net to the OU-Net configuration, as detailed in Table 2. The substitution of standard skip connections with offset convolution initially precipitated a notable decline in performance metrics. Specifically, the mean IoU decreased from 36.33% to 26.20%, and the mean F1-score dropped from 53.30% to 41.51%. These results indicate that while offset convolution possesses the capacity to alter spatial sampling, its unguided application results in severe under-segmentation, thereby causing the model to fail in capturing significant portions of the target structure.

Subsequent improvements were observed with the MDOAU-Net architecture, which incorporated the multi-scale feature fusion module alongside a standard attention mechanism. This integration effectively reversed the performance degradation observed in the OU-Net. The mean IoU recovered to 37.84%, thereby surpassing the original U-Net baseline. Furthermore, experiments evaluating individual modules showed that simply adding these components to the baseline without proper structural synergy led to sub-optimal performance. This evidence suggests that the synergy between multi-scale context and attention mechanisms in MDOAU-Net empowers the model to discriminate between the shipwreck and the background with greater efficacy.

The proposed model, SW-Net, delivered the best overall results. After replacing the conventional attention mechanism with the offset convolution and the DAM, the model reached the highest mean IoU of 39.43% and a mean F1-score of 56.56%. Its effectiveness is also reflected in the high stability across multiple independent runs, recording a consistently low standard deviation. The statistical results indicate that the DAM helps guide offset convolution so that the network can pay closer attention to shipwreck structures without losing important spatial details. This allows SW-Net to better balance missed detections and false alarms, resulting in more accurate, stable, and reliable segmentation.

To further evaluate the necessity of the proposed structural constraints against unguided feature fitting capabilities, a comparative analysis of the baseline U-Net, the OU-Net, the MDOAU-Net, and the SW-Net was conducted. In general deep learning applications, unconstrained trainable spatial parameters are believed to offer superior feature fitting capabilities. This unconstrained approach is represented by the OU-Net variant, which relies entirely on unguided deformable operations. However, the experimental data reveals that this configuration causes a severe performance drop, yielding an IoU of only 26.20% and an F1-score of 41.51%. This significant degradation demonstrates that overly flexible spatial sampling mechanisms easily overfit the severe speckle noise inherent in side-scan sonar imagery. To resolve this issue, the proposed SW-Net does not freeze the network weights but instead introduces a fixed feature stacking method. By fusing four features around each pixel for analysis in a predefined spatial layout, the model effectively constrains the deformable sampling process. This fixed structural guidance prevents the network from being misled by acoustic interference. Consequently, the SW-Net achieves the highest IoU of 39.43% and an F1-score of 56.56%. This comparative experiment demonstrates the necessity of utilizing a fixed feature stacking method to ensure robust geometric perception in noisy acoustic environments.

### 4.6. Sensitivity Analysis

This section analyzes the impact of key hyperparameters on the performance of the proposed SW-Net. Comparative experiments were conducted to determine the optimal settings for three specific parameters, namely, the Gaussian kernel size, the directional dimension, and the channel reduction ratio. The following analysis evaluates how variations in these parameters influence segmentation accuracy, focusing on the trade-off between feature preservation and noise suppression to identify the most effective configuration for shipwreck detection.

#### 4.6.1. Sensitivity of Kernel Size

The kernel size sensitivity analysis shows clear differences in how SW-Net extracts features and performs segmentation. The statistical results presented in Table 3 indicate that the model’s performance does not scale linearly with the kernel size. Initially, smaller kernel sizes like 3 and 5 yield competitive mean IoUs of 38.34% and 38.17%, respectively. However, when the kernel size is increased to 7, the model achieves its optimal balance, recording the highest mean IoU of 39.43% and a mean F1-score of 56.56%. The consistently low standard deviation at this scale further demonstrates that this superior performance is highly stable across multiple independent runs, effectively ruling out the influence of random initialization.

At the boundaries of the tested kernel size range, the model exhibited markedly different behaviors. While a kernel size of 3 captures local details reasonably well, it falls short of the optimal contextual understanding provided by a size of 7. Conversely, further enlarging the receptive field introduces significant performance fluctuations. Notably, at a kernel size of 9, the mean IoU drops to its minimum of 36.00%. This sharp decline underscores that an excessively large or improperly scaled receptive field may incorporate excessive background noise, thereby weakening the local details needed for accurate target recognition. Although the performance slightly recovers at a kernel size of 11, it still underperforms compared to the optimal setting. Overall, the experimental results indicate that a kernel size of 7 achieves superior performance, as it offers the strongest balance between capturing sufficient spatial context for detecting shipwreck targets and avoiding the inclusion of excessive acoustic interference.

#### 4.6.2. Sensitivity of Directional Dimension

The sensitivity analysis concerning the directional dimension parameter elucidates how the complexity of spatial sampling affects segmentation performance. The statistical results presented in Table 4 reveal that the model achieves its peak performance when configured with 8 directional dimensions, recording the highest mean IoU of 39.43% and a mean F1-score of 56.56%. Interestingly, the configuration utilizing 2 directional dimensions demonstrates highly competitive results by achieving a mean IoU of 39.42% and an F1-score of 56.54%. Alongside these high metrics, this specific setting also exhibits the lowest standard deviation, recording merely 0.31% for the IoU and 0.32% for the F1-score. This indicates that a lower dimensionality can provide an exceptionally stable and precise feature representation, effectively minimizing variance across multiple independent runs.

In contrast, other configurations introduce noticeable performance fluctuations. When the dimension is set to 4, the mean IoU drops to 37.78%. Furthermore, increasing the dimensionality to 16 results in the poorest performance across all metrics, yielding the lowest mean IoU of 36.49% alongside a minimum mean F1-score of 53.47%. This significant drop suggests that an inappropriate number of directional dimensions may excessively fragment the feature space, leading to a loss of valid target details and making the model more susceptible to acoustic noise. Although the performance rebounds at a dimension of 32 by reaching a mean IoU of 39.08%, it still does not surpass the peak achieved at dimension 8. Ultimately, while the 2-dimension setting serves as a highly robust configuration with minimal variance, the 8-dimension configuration provides the optimal balance for maximizing overall segmentation accuracy.

#### 4.6.3. Sensitivity of Channel Reduction Ratio

The analysis of the channel reduction ratio shows that a moderate reduction factor provides the best trade-off between feature representation and segmentation performance. In particular, the model configured with a reduction ratio of 4 achieves the best overall results as presented in Table 5. This configuration records the highest mean IoU of 39.43% alongside the maximum mean F1-score of 56.56%. These results suggest that reducing the channel dimension by a factor of 4 effectively retains important spatial information while removing unnecessary feature redundancy.

In contrast, a reduction ratio of 1 yields weaker results by producing the lowest mean IoU of 37.40% and a minimum mean F1-score of 54.44%. This indicates that without any channel reduction, the model retains excessive noise in the feature maps, which ultimately reduces segmentation precision. When the reduction ratio is increased to 16, the model becomes notably more conservative. Although this high reduction ratio exhibits the lowest standard deviation for both the IoU at 0.32% and the F1-score at 0.34%, its mean performance drops to an IoU of 38.27%. This pattern implies that an excessive channel reduction removes the important fine details needed to detect subtle shipwreck structures. Therefore, a reduction ratio of 4 is identified as the optimal setting, as it provides the strongest balance between accurate target detection and the suppression of background errors.

#### 4.6.4. Summary of Sensitivity Analysis

The comprehensive sensitivity analyzes conducted on the kernel size, the directional dimension, and the channel reduction ratio collectively elucidate the influence of hyperparameter configuration on the network’s predictive capability. It is observed that variations in these structural parameters lead to discernible fluctuations in segmentation metrics, which indicates that specific configurations are necessary to maximize the trade-off between target feature aggregation and background noise suppression. Although changing the parameter settings affects the exact numerical results, the performance of the SW-Net remains consistently high and remarkably stable. Even under less optimal settings, the segmentation accuracy achieved by the proposed model generally exceeds that of the baseline models used for comparison. This consistent advantage suggests that the core architectural design of SW-Net provides a resilient foundation for semantic segmentation tasks that is not overly dependent on precise hyperparameter tuning.

### 4.7. Model Complexity and Inference Efficiency

Based on the comparison of model parameter size and computational speed, the results show that the SW-Net achieves a better balance between efficiency and performance than the other models. As listed in Table 6, the traditional U-Net, SegNet, Attention U-Net, and TriEncoderNet all require relatively high computational cost or memory footprints. Among them, the TriEncoderNet has the heaviest computational burden with 79.07 million parameters and 319.37 G (Giga) floating-point operations, followed by the Attention U-Net with 34.88 million parameters and 266.23 G floating-point operations. While the SegNet reduces the computation to 160.22 G floating-point operations, it still has a large parameter count of 29.44 million.

A significant improvement in efficiency is observed with the UNet++, which drastically reduces the parameter count to 9.16 million and the computational cost to 139.46G floating-point operations, all while maintaining a competitive IoU of 36.82%. Further optimization is evident in the MDOAU-Net and LHNet, which lower their parameters to 4.09 million and 4.95 million respectively, though LHNet experiences a significant drop in segmentation accuracy.

However, the best overall results are achieved by the SW-Net. This model requires the fewest resources, utilizing only 4.01 million parameters and 41.45 G floating-point operations. Despite being the most lightweight and computationally efficient architecture among those tested, the SW-Net simultaneously attains the highest segmentation accuracy with an IoU of 39.43%. These findings indicate that the SW-Net effectively minimizes model complexity and computational demand without compromising segmentation performance, making it highly suitable for applications where computational resources are limited.

To comprehensively evaluate the feasibility of real-time deployment on autonomous underwater vehicles, actual inference latency and FPS tests were conducted to move beyond theoretical metrics like floating-point operations and parameter counts. As presented in Table 6, the proposed SW-Net achieves an average inference time of 21.95 ms, which translates to an operating speed of 45.58 FPS.

While lightweight architectures such as SegNet exhibit slightly faster inference speeds, they suffer from a significant degradation in segmentation accuracy. Conversely, compared to well-performing models like MDOAU-Net and UNet++, SW-Net not only achieves the highest IoU but also demonstrates faster inference speeds. The recently introduced TriEncoderNet, despite its competitive accuracy, suffers from a severe bottleneck in inference speed, achieving only 8.62 FPS. The high efficiency of SW-Net is largely attributed to the fixed-weight design of the directional filter bank and the parameter-free asymmetric padding of the offset convolution. These specific structural designs enhance feature extraction without introducing substantial computational overhead during inference.

Considering the physical constraints of autonomous underwater vehicle operations, cruising speeds are relatively slow. Furthermore, side-scan sonar data acquisition rates are typically lower than standard optical video frame rates. Consequently, a processing speed of approximately 45 FPS is highly adequate for real-time onboard target detection and survey guidance. These results validate that SW-Net successfully strikes an optimal balance between segmentation accuracy, parameter scale, and actual inference efficiency.

## 5. Discussion

### 5.1. Effectiveness of Directional Priors and Error Analysis

The experimental results validate the central hypothesis of this study that embedding physical directional priors into deep learning architectures can improve shipwreck segmentation in side-scan sonar imagery. Unlike standard CNNs that treat spatial directions uniformly and may overfit to seabed speckle noise, SW-Net explicitly extracts geometric regularities through the non-trainable directional filter bank. The ablation study further shows that this directional guidance is important for the offset convolution module. When offset convolution is used without sufficient directional constraint, the model suffers from severe under-segmentation, whereas the full SW-Net achieves the best performance with an IoU of 39.43% and an F1-score of 56.56%.

From the perspective of classical pattern recognition, the remaining errors of SW-Net can be understood as two types. Type I errors correspond to false positives, where non-shipwreck regions are incorrectly segmented as shipwrecks. In this study, such errors mainly occur in areas containing highly reflective rocks, sand ripples, or strong acoustic shadows, because these structures may produce echo patterns similar to shipwreck debris. Type II errors correspond to false negatives, where real shipwreck pixels are missed by the model. These errors mainly occur when shipwrecks are partially buried, located in the nadir gap, or fragmented by long-term corrosion and sediment accumulation.

This error-type analysis indicates that further progress should not rely only on increasing network complexity. To reduce Type I errors, future work may incorporate bathymetric data, magnetometer readings, or multi-frequency sonar imagery to distinguish artificial targets from rock-like geological features. To reduce Type II errors, temporal or multi-view sonar sequences may be used to recover weak target boundaries and improve the continuity of fragmented shipwreck structures. Therefore, integrating physical priors with multi-modal or sequential information may be a more promising direction for automated marine archaeology.

The lightweight design of SW-Net also supports its practical use in resource-constrained underwater platforms. By offloading part of the edge extraction process to fixed signal-processing filters, the model achieves the highest segmentation accuracy among the evaluated methods while requiring only 4.01 million parameters. Its inference speed of 45.58 FPS further suggests that real-time “search-and-inspect” missions on autonomous underwater vehicles are feasible.

### 5.2. Analysis of Task Difficulty and Performance Limits

Although the proposed SW-Net achieves the SOTA performance on the shipwreck dataset with a mean IoU of 39.43% and a mean F1-score of 56.56%, these absolute metric values are noticeably lower than those typically observed in conventional optical image segmentation tasks. It is crucial to objectively analyze the upper limit of task difficulty in side-scan sonar imagery to understand these metrics in their proper context.

The primary factor limiting the theoretical upper bound of segmentation accuracy is the inherent physical nature of acoustic imaging. Side-scan sonar images are heavily degraded by speckle noise, acoustic shadows, and multipath reverberations. Unlike optical images that provide clear texture and color features, sonar images are monochromatic and exhibit extremely low contrast between the target and the background. Shipwrecks, especially those that have been submerged for long periods, often suffer from structural collapse and biological fouling, causing them to blend seamlessly into the surrounding seabed.

This extreme degradation leads to severe label ambiguity at the target boundaries. In conventional optical datasets, human annotators can achieve an inter-annotator agreement (often measured as human-level IoU) of over 90%. However, in complex sonar imagery, the transition zone between a shipwreck and the seabed is highly blurred. If multiple human experts were to independently annotate the exact pixel boundaries of these shipwrecks, the inter-annotator IoU would likely be severely capped, theoretically limiting the maximum achievable IoU for any deep learning model to a much lower threshold (e.g., around 50% to 60%) compared to optical tasks.

Furthermore, shipwrecks typically occupy a very small fraction of the total image area, leading to a severe class imbalance. In such heavily imbalanced and noisy scenarios, any slight deviation in boundary prediction or a small cluster of false-positive pixels caused by seabed reverberations will drastically penalize the IoU and F1-score. Therefore, while an IoU of 39.43% may seem modest in the broader field of computer vision, it represents a highly competitive and practically valuable achievement in the specialized domain of automated shipwreck detection in side-scan sonar imagery. Future research aiming to push this upper limit will likely need to incorporate acoustic physical priors or utilize multi-view sonar sequences rather than relying solely on spatial pixel features.

Overall, the results indicate that incorporating physical and geometric priors into deep learning models is a useful direction for improving the interpretation of acoustic remote sensing data.

## 6. Conclusions

This study introduces SW-Net, a deep learning framework designed for the automated detection and segmentation of shipwrecks in SSS imagery, and evaluates its performance through a series of experiments. To address common challenges such as heavy speckle noise, target deformation, and severe class imbalance, a tailored encoder–decoder architecture was proposed to bridge the gap between signal processing priors and deep feature learning.

The core contribution of this work lies in the integration of a non-trainable DFB, a DAM, and a structurally guided offset convolution module. By incorporating physical knowledge of edge direction into the feature extraction, the model becomes more responsive to man-made structures while reducing interference from seabed reverberation. Based on experiments conducted on the AI4Shipwrecks dataset, SW-Net outperformed the evaluated competing models, achieving an F1-score of 56.56% and an IoU of 39.43%, thereby reaching a state-of-the-art level. Notably, it required relatively low computational cost, making it computationally efficient and suitable for resource-constrained underwater platforms. The results also show that designing a tailored framework with modules such as the DFB can help balance segmentation accuracy and computational efficiency, which makes it suitable for practical underwater detection and cultural heritage preservation. Overall, this work contributes to current image segmentation research, especially in the field of shipwreck detection.

In addition, the findings show that embedding geometric constraints into deep networks effectively tackles the challenges of low-contrast sonar data. Future work may proceed in two main directions. One is adapting the model for deployment on embedded systems to evaluate its performance under real-time survey conditions. The other is exploring multi-modal approaches, such as combining bathymetric data or multi-frequency sonar imagery, to better distinguish shipwrecks from complex geological features.

## Figures and Tables

**Table 1:** Quantitative comparison of different segmentation models on the shipwreck SSS dataset.

Model	IoU		F1-Score	
	Mean ↑	Std	Mean ↑	Std
U-Net	36.33%	0.39%	53.30%	0.42%
SegNet	32.63%	0.23%	49.20%	0.26%
Attention U-Net	36.39%	0.46%	53.36%	0.50%
UNet++	36.57%	0.41%	53.56%	0.44%
MDOAU-Net	37.84%	0.45%	54.90%	0.48%
LHNet	34.37%	0.30%	51.15%	0.33%
TriEncoderNet	37.39%	0.27%	54.43%	0.28%
SW-Net	**39.43%**	0.47%	**56.56%**	0.48%

↑ indicates higher is better, and the bolded data represent the highest values.

**Table 2:** Ablation study on the effects of different modules on segmentation performance.

Model	IoU		F1-Score	
	Mean ↑	Std	Mean ↑	Std
U-Net	36.33%	0.39%	53.30%	0.42%
OU-Net	26.20%	0.23%	41.51%	0.28%
MDOAU-Net	37.84%	0.45%	54.90%	0.48%
U-Net + DFB	35.22%	0.15%	52.09%	0.17%
U-Net + DAM	33.64%	0.50%	50.35%	0.56%
SW-Net	**39.43%**	0.47%	**56.56%**	0.48%

↑ indicates higher is better, and the bolded data represent the highest values.

**Table 3:** Comparison of model performance with varying kernel sizes.

Kernel Size	IoU		F1-Score	
	Mean ↑	Std	Mean ↑	Std
3	38.34%	0.52%	55.42%	0.54%
5	38.17%	0.44%	55.25%	0.46%
7	**39.43%**	0.47%	**56.56%**	0.48%
9	36.00%	0.47%	52.93%	0.51%
11	37.82%	0.45%	54.88%	0.47%

↑ indicates higher is better, and the bolded data represent the highest values.

**Table 4:** Comparison of different directional dimensions on segmentation performance.

Directional Dimension	IoU		F1-Score	
	Mean ↑	Std	Mean ↑	Std
2	39.42%	0.31%	56.54%	0.32%
4	37.78%	0.42%	54.84%	0.44%
8	**39.43%**	0.47%	**56.56%**	0.48%
16	36.49%	0.49%	53.47%	0.53%
32	39.08%	0.36%	56.19%	0.38%

↑ indicates higher is better, and the bolded data represent the highest values.

**Table 5:** Comparison of different channel reduction ratios on segmentation performance.

Channel Reduction Ratio	IoU		F1-Score	
	Mean ↑	Std	Mean ↑	Std
1	37.40%	0.36%	54.44%	0.38%
4	**39.43%**	0.47%	**56.56%**	0.48%
16	38.27%	0.32%	55.35%	0.34%

↑ indicates higher is better, and the bolded data represent the highest values.

**Table 6:** Quantitative comparison of segmentation performance, parameter scale, and computational cost across different network architectures.

Model	IoU ↑	Number of Parameters (M) ↓	FLOPs (G) ↓	Inference Time (ms) ↓	FPS ↑
U-Net	36.33%	31.04	218.65	22.67 ± 0.69	44.12 ± 0.39
SegNet	32.63%	29.44	160.22	**18.60 ± 0.61**	**53.78 ± 0.50**
Attention U-Net	36.39%	34.88	266.23	31.27 ± 0.88	31.98 ± 0.26
UNet++	36.57%	9.16	139.46	24.45 ± 0.80	40.92 ± 0.39
MDOAU-Net	37.84%	4.09	59.48	26.41 ± 0.93	37.89 ± 0.38
LHNet	34.37%	4.95	73.15	31.85 ± 0.46	31.4 ± 0.82
TriEncoderNet	37.39%	79.07	319.37	116.31 ± 0.11	8.62 ± 0.32
SW-Net	**39.43%**	**4.01**	**41.45**	21.95 ± 0.76	45.58 ± 0.45

↑ indicates higher is better, ↓ indicates lower is better, and the bolded data represent the highest values.

## Data Availability

The data presented in this study are openly available in Baidu Net disk at https://pan.baidu.com/s/1Z6itXnX4mlcVmmGl40_Drg?pwd=8dfn, accessed on 15 May 2026.

## Abbreviations

The following abbreviations are used in this manuscript:

SSS	Side-scan sonar
AUV	Autonomous underwater vehicle
DFB	Directional filter bank
DAM	Directional attention mechanism
IoU	Intersection over union
FPS	Frames per second
TP	True positive
FP	False positive
TN	True negative
FN	False negative
std	standard deviation
